# Effects of an 8-week Baduanjin intervention combined with low-carbohydrates diet among overweight people who struggle with drug addiction

**DOI:** 10.3389/fpubh.2022.989519

**Published:** 2022-10-21

**Authors:** Dongming Jia, Yuming Xu

**Affiliations:** ^1^School of Public Health, Hangzhou Normal University, Hangzhou, China; ^2^Zhejiang Police Vocational College, Hangzhou, China; ^3^School of Physical Education, Hangzhou Normal University, Hangzhou, China

**Keywords:** overweight, low-carbohydrate diet, Baduanjin, addicts, quality of life

## Abstract

**Background:**

Prior studies have consistently revealed that a combination of physical activity with caloric restriction results in a reduction in body weight in the general population. Both overweight and drug abuse are risk factors for poor physiological health, and poor mental health has been associated with drug abuse and unhealthy body mass index (BMI). However, the effects of low-carbohydrates diet (LC) combined with mind-body exercise intervention on improving anthropometric characteristics, lipid metabolism, quality of life, and craving among overweight people who struggle with drug addiction have yet to be clarified.

**Methods:**

Fifty-four eligible male patients were randomly assigned to the control group (CON; *n* = 18), the Baduanjin intervention group (BA, 60 min × 5 times/week, 8 weeks; *n* = 18), and the Baduanjin combined with LC intervention group (LC; *n* = 18). We compared the anthropometric characteristics, blood biochemical parameters, quality of life, and drug craving responses between the three groups at baseline (week 0), week 4, and week 8.

**Results:**

After repeated measurements in the general linear model, both the BA and LC groups exerted significant effects on decreasing waist circumference, BMI, body weight, hip circumference, body fat percentage, total cholesterol level, and triglyceride level (*P* < 0.05). There was no significant difference in the HDL-C level observed among the BA and LC groups at 8 weeks (*P* > 0.05); however, there was an overall upwards trend. A significant change in trends in the quality of life scale score was determined in the three groups (*P* < 0.001). The three groups showed reductions in visual analog scale score distribution over time (*P* < 0.05).

**Conclusions:**

Either Baduanjin or an 8-week Baduanjin combined with LC can significantly reduce anthropometric characteristics and body composition, enhance the quality of life, and reduce craving in overweight/obese patients. Baduanjin combined with LC is particularly effective in improving lipid metabolism.

## Introduction

Drug addiction is a type of mental illness that affects a significant number of people, with an estimate of nearly 1.4 million in China (1). Most patients received rehabilitation interventions in drug rehabilitation centers (DRCs). The Chinese government has implemented the Healthy China Action Plan (2019–2030), which aims to provide health services to every citizen by 2030, including managing addiction as a chronic condition (2). Drug-seeking and drug-taking are the main characteristics of drug addiction (3). Depending on the type, dose and frequency of drug abuse and preexisting health status, drug use may have a variety of short- and long-term effects on health and society, such as heart attacks, stroke, crime, violence, changes in blood pressure, medical expenses, mental health problems, overuse, and ultimately death (4). Indeed, the US National Institute on Drug Abuse (NIDA) recommended that addiction treatment outcome research seek to measure a number of domains, specifically craving, quality of life (QOL), psychosocial functioning, self-efficacy, and social support (5). However, addiction research lags behind other biomedical fields in terms of evaluating QOL outcomes. In this article, we adopt National Institutes of Health's advice to reduce stigma and negative bias when talking about addiction: we use “people who struggle with drug addiction/patient” instead of “addict/drug abuser” (6).

Although various drug-dependent treatments have been developed, including pharmacological, psychological, and sociological interventions, their efficacy is limited. The side effects of pharmacological therapy on the physical and psychological health of users should be noted. Other non-pharmacological treatments have been developed, such as contingency management, cognitive–behavioral therapy, and repetitive transcranial magnetic stimulation (7). In addition to these approaches, exercise has been indicated as a safe and non-invasive non-pharmacological treatment, either in isolated exercise-based interventions or in combination with other addiction treatment strategies (8).

In our previous survey, we found that 1 year after rehabilitation in DRC, more than 1/3 of addicts were overweight, and there was an upwards trend, although early studies have found that more than 60% of patients have multiple nutritional deficiencies (9). Overweight and obesity are risk factors for various chronic diseases, such as diabetes, hypertension, and cardiovascular disease (10). Compared to the general population, patients with drug addiction have an increased risk of additional hazardous lifestyles and suffer from more chronic diseases, adding to their already significantly higher morbidity and mortality (11). Many studies have shown the benefits of dietary changes, in addition to physical activity, for overweight and obese individuals (12). A meta-analysis indicated that low-carbohydrate diet (LC) exert a more significant long-term effect on weight loss than the traditional low-fat diet (13). LC can lead to quick weight loss and become part of a healthy lifestyle to prevent cognitive decline. LC (60–150 g of total carbohydrate per day) as an intervention can be used as an effective dietary strategy to lose weight and manage the pathological status of chronic diseases (14–16). Notably, previous studies on LC intervention focused on other chronic diseases rather than drug addiction. Researchers and clinicians should pay more attention to these people.

As one of the traditional Chinese health-promoting exercises (TCE, e.g., Tai Chi, Baduanjin, Wuqinxi), Baduanjin has eight simple postures and movements, which makes it readily accessible to a variety of people (17). Previous studies on TCE in drug rehabilitation suggest that long-term TCE is beneficial to improving physical and mental health, but few studies have involved Baduanjin (7, 18). Many studies have examined the effects of Baduanjin on different aspects of health among the general population, including mental health, cardiovascular parameters, quality of life, sleep quality, cardiorespiratory fitness, physical performance, balance, and flexibility (19). A systematic review included 47 studies with a combined total of 3,877 participants, and two reported adverse events. The risk of harm from Baduanjin exercise may be minor, as suggested by a systematic review, and older adults or patients with chronic illness are more likely to experience benefits associated with its clinical effects and affordability (20). A proof-of-concept study in Parkinson's disease suggests that Baduanjin intervention by telerehabilitation seems feasible with no major technical issues or adverse events and high adherence, which is acceptable (patient satisfaction) (21). Recent studies have used Baduanjin exercise as an adjuvant therapy and rehabilitation method for patients with COVID-19 (22). During the epidemic period, the 12-week Baduanjin exercise can alleviate the anxiety of college students about COVID-19, decrease the prevalence of low back pain, and enhance their psychological wellbeing (23). A review study on the beneficial effects of Baduanjin found that it may be more suitable for populations with physical or cognitive impairments (24). Additionally, one study reported exercise mode heterogeneity among studies of Baduanjin and pointed out that most Baduanjin research reports bear ambiguities rendering the methods non-reproducible, and it needs adequate description if the exercise involves an intention to regulate breathing or mental activities (25).

Traditional Chinese health-promoting exercise, as an exercise intervention for drug addiction, can promote the physical and mental health of people with drug addiction. Collectively, the combination of LC and exercise training seems to also be beneficial for this special population. Hence, to fill this knowledge gap, we aimed to investigate the safety, effectiveness, and craving among overweight people who struggle long-term with drug addiction practicing Baduanjin and who have adopted LC. Thus far, no research has been conducted on the effect of Baduanjin and nutritious diet on the health-related outcomes (e.g., anthropometric characteristics, physical fitness, lipid metabolism, quality of life, and craving response). We also attempted to explain the relationships between overweight and drug abuse and offer practical strategies in exercise intervention and health management of rehabilitation for drug addiction. With these basic considerations in mind, we conducted a randomized controlled trial for 8 weeks to compare the effects of (i) 8 weeks of Baduanjin and (ii) Baduanjin with LC on anthropometric assessments and body composition, blood biochemical parameters, quality of life, and craving response in overweight patients. Based on the reviewed literature, the following hypotheses will be tested: as an intervention, Baduanjin combined with LC among overweight people who struggle with drug addiction was assumed to induce a more significant loss in body weight (BW), BMI, BFP and craving response and better QOL and lipid metabolism outcome than Baduanjin alone.

## Materials and methods

### Participants

Before recruitment, the sample size was calculated using GPower (Version 3.1). With a 20% potential dropout rate considered, the goal was to recruit 54 participants in total for three groups (power = 0.8 at α = 0.05; effect size = 0.4). A sample size of 18 participants for each group was necessary.

We preliminarily evaluated 60 male patients in a DRC in China between December 2021 and February 2022. All participants had stayed in the DRC for more than six months within the study period. The inclusion criteria were as follows: (1) age 18–60 years; (2) illegal drug-dependent use (assessed by the Chinese version of the Addiction Severity Index) (26); (3) BMI ≥24 kg/m^2^; (4) no cognitive impairment and ability to complete the questionnaire independently; (5) no serious diseases that prevent participation in physical activities; (6) not undergoing psychotropic drug treatment; and (7) no medical or neurological diseases or trauma of the central nervous system.

**Figure 1:**
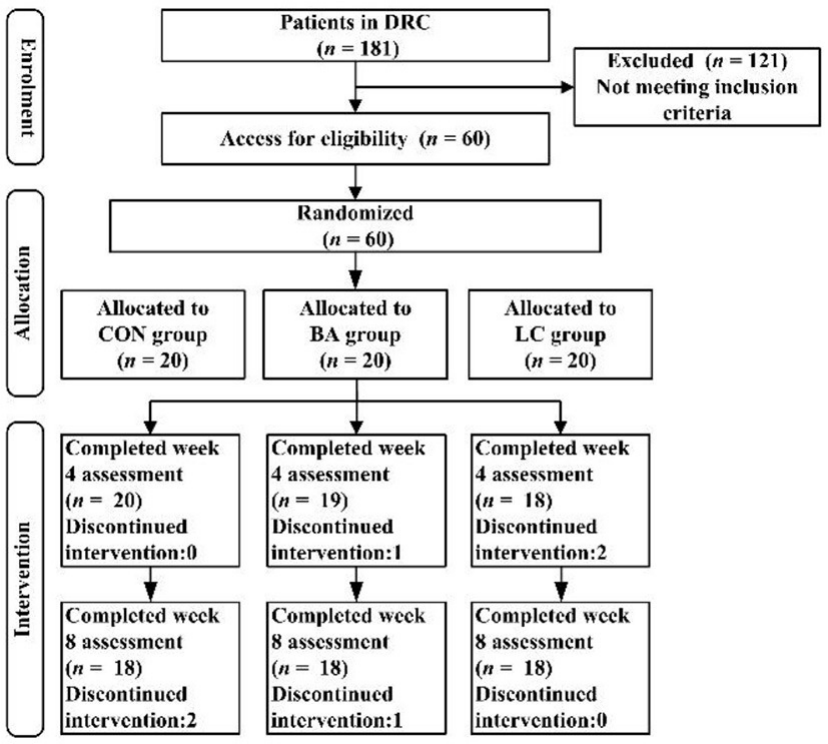
Flowchart of the study. CON, general physical activity; BA, Baduanjin intervention; LC, Baduanjin combined with LC intervention.

The study was conducted in accordance with the Declaration of Helsinki. The research protocol was approved by the Ethics Committee of Zhejiang Police Vocational College (No. ZJRP-1025/2021). Participation in the study was entirely voluntary, and informed consent was obtained from each qualified participant. To ensure that the sample would be significantly representative and to reduce statistical error, we adopted a stratified sampling methodology to collect the relevant data. In the first sampling stage, we stratified all samples according to age, type of drug, and drug use duration. Then, the participants were randomly assigned to three groups in a 1:1:1 ratio by using PASW software (Release 22.0; IBM, New York, United States): (i) general physical activity (CON, *n* = 20), (ii) Baduanjin intervention (BA, *n* = 20), and (iii) Baduanjin combined with LC intervention (LC, *n* = 20). Six participants dropped out because of schedule conflicts or personal reasons. Ultimately, 54 participants completed the project, and all pre- and postmeasurements were included in the final data analysis (Figure 1). The coaches or researchers recorded any intervention-related adverse events reported by the participants.

### Exercise intervention

We used the Compendium of Physical Activities to estimate the exercise intensity for general physical activity (CON) and Baduanjin intervention (BA) in metabolic equivalents (METs). General physical activity is categorized as light-intensity activity with an average of 2.7 METs; Baduanjin with ~4.8 METs is considered moderate-intensity activity.

Participants in the BA and LC groups underwent training for Baduanjin (the version published by the State General Administration of Sport of China in 2003). The training was conducted under the supervision of two trained coaches for 8 weeks (5 days/week, four training sessions for a total of ~60 min/day). Each training session consisted of eight movements: (1) Double Hands Hold Up the Heavens, (2) Left–Right Opening the Bow, (3) Single Lift, (4) Five Weaknesses and Seven Injuries, (5) Sway the Head and Swing the Tail to Get Rid of the Heart Fire, (6) Two Hands Hold the Feet, (7) Screw the Fist with Fiery Eyes, and (8) Seven Disorders and Hundreds of Illnesses Disappear (27). The coaches recorded participant attendance to measure adherence.

### Diet intervention

In this study, only participants in the LC group underwent the 8-week Baduanjin with LC intervention. The daily calorie intake before and after the intervention was ~2,000–2,700 kcal. The nutritional requirements of LC adopted that fats, proteins, and carbohydrates comprised ~65, 25, and 10% of their daily energy intakes, respectively. Carbohydrates were limited to 70–130 g/day. All participants in the CON and BA groups consumed a nutritionally balanced diet of 1,800–2,400 kcal/day (15% calories from protein and 30% calories from fat) during the study.

To support treatment fidelity, Baduanjin intervention focused on improving physical function and mental health, and LC intervention focused on reducing body weight. Each participant will be given a Baduanjin manual with exercise instructions and safety information. Participants in the LC group received dietary recommendation flyers according to the guidelines of the China Society for Nutrition to reduce their body weight. Questions from participants about dietary methods were immediately answered by the researchers throughout the experiment. The coaches and researchers passed fidelity assessment prior to starting the exercise intervention and diet intervention for the current study and maintained fidelity for the duration of the study.

### Outcome measures

Outcome measures were obtained at baseline (week 0) and after weeks 4 and 8 to evaluate changes in anthropometric characteristics, blood biochemical parameters, quality of life, and drug craving response among overweight male patients from the three intervention groups.

### Anthropometric assessments and body composition

Waist circumference (WC) between the rib frame and the ilium was measured as the participant exhaled gently. WC was measured with an inelastic tape on bare skin, accurate to 0.1 cm between the lowest ribs and the upper edge of the iliac crest. Hip circumference (HC) was measured over the widest part of the buttocks. BMI was calculated as weight (kg) divided by height squared (m^2^), and the values were accurate to 0.1 cm and 0.1 kg, respectively. BFP was determined using body composition analysers. Each index (WC, BW, BMI, HC, and BFP) was measured within the same morning and evaluated by the same researchers before and after the intervention. Overweight and obesity were identified as BMI 24–28 kg/m^2^ and BMI equal to or over 28 kg/m^2^, respectively, according to the Working Group on Obesity in China (WGOC) (28).

### Secondary outcomes

#### Blood biochemical parameters

Blood sampling was conducted in the morning after an overnight fast. Parameters of lipid metabolism, including total cholesterol (TC), high-density lipoprotein-cholesterol (HDL-C), and triglyceride (TG) levels, were collected (Baver ADVIA2400, Germany).

#### Quality of life evaluation

The Quality of Life Scale for Drug Addiction (QOL-DA) was developed by Chonghua Wan in 1997 for drug-dependent patients in China to measure their quality of life (29). The scale consisted of four dimensions and 40 items, with the physical, psychological, social and symptom/toxicity domains containing 9, 9, 11, and 11 items, respectively. Each item was assigned a score of 5, and the total score ranged from 40–200 points. Test–retest reliability in the physical function, psychological function, social function, and toxicity domains were 0.82, 0.64, 0.78, and 0.76, respectively (29). A low score was closely related to poor quality of life.

#### Drug craving response

The Visual Analog Scale (VAS) of cravings was used to evaluate the participants' current level of drug cravings. The VAS was denoted by a line segment, with two ends set to 0 (no craving at all) and 10 (the strongest craving in the imagination). The research subjects were required to mark the corresponding position of the line segment according to the subjective craving of the participants during the investigation. The distance between the site and Point 0 was the craving score (30); the higher the score, the higher the current drug craving. Currently, VAS is the most commonly used tool, with good reliability and validity. To assess subjective craving, the Cronbach's alpha coefficient of the VAS in the present study was 0.87 (31).

### Statistical analysis

Statistical data were analyzed using PASW software (Version 18, SPSS, Inc., IBM, Chicago, IL, United States). Pearson's chi-squared test was applied for demographic (categorical) variables. Normal distribution tests were employed for continuous variables. The repeated-measures analysis of continuous numerical variables was conducted using the general linear model repeated measures. Two classified variables were analyzed using a generalized estimating equation (GEE), and a paired chi-square test was used to assess the predictive accuracy of the model. The time point (baseline, 4 weeks and 8 weeks) was the within-group factor, the groups (CON, BA, and LC) were the intergroup factors, and the type of drug (heroin, meth) was the covariate. The effects of intervention, drug type, and time point on the outcome indexes (LBM, BMI, WC, HC, BFP, TC level, HDL-C level, TG level, QOL-DA, and VAS) were determined. If the time point–group interaction was significant, the simple effect between the time point and the intervention was further investigated with the *post hoc* SIDAK correction. Statistical significance was set at *P* < 0.05.

## Results

### Demographic characteristics of the participants

All eligible participants (*n* = 60) were randomly assigned to the control (*n* = 20), BA (*n* = 20) and LC (*n* = 20) groups. A total of 54 participants ultimately completed the 8-week experiment and thus were included in the data analysis; the dropout rate was 10%. The demographic characteristics of the included participants and the data comparison between the three groups at baseline are listed in Table 1. The CON, BA and LC groups had a mean BW of 78.0 ± 6.99, 76.5 ± 8.07, and 81.5 ± 5.80 kg, respectively, corresponding to a BMI of ~27.0 kg/m^2^. There were no significant differences in age, BW, WC, HC, BFP, TC level, HDL-C level, TG level, marital status, educational level, occupation, type of drug, or drug use duration among the three groups (*P* > 0.05). A significant difference in BMI (*P* < 0.05) was reported. BMI was included as a main effect term in all GEE models to control for its confounding effect.

**Table 1:** Demographic data of the participants on admission (*N* = 54).

**Characteristic**	**CON (*n* = 18)**	**BA (*n* = 18)**	**LC (*n* = 18)**	***F*/χ^2^**	***P***
Mean age (SD), year	35.6 (3.62)	36.4 (3.55)	37.1 (3.68)	0.775	0.466
Mean BW (SD), kg	78.0 (6.99)	76.5 (8.07)	81.5 (5.80)	2.418	0.099
Mean BMI (SD), kg/m^2^	27.9 (2.74)	26.6 (1.61)	28.6 (1.51)	4.678	**0.014**
Mean WC (SD), cm	96.3 (6.69)	93.9 (6.26)	98.2 (6.19)	2.028	0.142
Mean HC (SD), cm	107.3 (9.56)	106.7 (8.92)	108.4 (9.22)	0.157	0.855
Mean BFP (SD), %	29.3 (6.37)	28.9 (5.71)	30.5 (6.06)	0.341	0.713
Mean TC (SD), mmol/L	4.78 (0.76)	4.72 (0.82)	4.80 (0.88)	0.046	0.955
Mean HDL-C (SD), mmol/L	1.26 (0.32)	1.27 (0.34)	1.25 (0.40)	0.014	0.986
Mean TG (SD), mmol/L	1.73 (0.82)	1.66 (0.75)	1.81 (0.85)	0.155	0.857
**Marital status**, ***n*** **(%)**				0.159	0.924
Married	7 (38.9)	7 (38.9)	6 (33.3)		
Single/divorced/widow	11 (61.1)	11 (61.1)	12 (66.7)		
**Educational level**, ***n*** **(%)**				0.444	0.801
High school or above	9 (50)	10 (55.6)	8 (44.4)		
Below high school	9 (50)	8 (44.4)	10 (55.6)		
**Occupation** ***n*** **(%)**				0.500	0.779
Unemployed	13 (72.2)	11 (61.1)	12 (66.7)		
Employed	5 (27.8)	7 (38.9)	6 (33.3)		
**Type of drug**, ***n*** **(%)**				0.172	0.918
Heroin	5 (27.8)	6 (33.3)	6 (33.3)		
Meth	13 (72.2)	12 (66.7)	12 (66.7)		
Drug use duration (SD), y	3.85 (0.71)	3.72 (0.68)	3.92 (0.80)	0.346	0.709

BW, body weight; BMI, body mass index; WC, waist circumference; HC, hip circumference; Meth, methamphetamine; CON, general physical activity; BFP, body fat percentage; TC, total cholesterol; HDL-C, high-density lipoprotein–cholesterol; TG, triglyceride; BA, Baduanjin intervention; LC, Baduanjin combined with LC intervention. A bold value of 0.014 means there is a difference (*p* < 0.05).

### Anthropometric characteristics and body composition

The time effect significantly influenced changes in WC (*F* = 34.146, *P* < 0.001), BMI (*F* = 45.373, *P* < 0.001), and BFP (*F* = 4.89, *P* < 0.011). However, no significant differences in BW and HC were found (*P* > 0.05; Table 2). Drug type exerted no significant effect on changes in BW, WC, BMI, or BFP (*P* > 0.05). Relative to those of the CON group from baseline to weeks 4 and 8, both the BA and LC groups showed modest reductions in BW, WC, BMI, and BFP (*P* < 0.05). No significant differences in group–time interaction analyses of BW, WC, BMI, and BFP were found between the groups (*P* < 0.05). This finding indicates that the changing trends in BW, WC, BMI, and BFP varied depending on the intervention. The BW, WC, BMI, and BFP were significantly reduced after intervention in the BA and LC groups. However, no significant differences in BW and HC existed (*P* > 0.05; Table 2).

**Table 2:** Changes in outcomes in the three groups (*N* = 54).

**Group**	**Time**	**BW (SD) (kg)**	**WC (SD) (cm)**	**BMI (SD) (kg/m^2^)**	**HC (SD) (cm)**	**BFP (SD) (%)**	**TC (SD) (mmol/L)**	**HDL-C (SD) (mmol/L)**	**TG (SD) (mmol/L)**	**QOL-DA (SD)**	**VAS Score < 5 *N* (%)**
CON (*n* = 18)	Week 0	78.0 (6.99)	96.3 (6.69)	27.9 (2.74)	107.3 (9.56)	29.3 (6.37)	4.78 (0.76)	1.26 (0.32)	1.73 (0.82)	110.8 (5.41)	9 (50.0)
	Week 4	79.8 (6.15)	98.4 (6.91)	28.4 (2.79)	108.2 (9.15)	30.2 (7.12)	4.75 (0.82)	1.24 (0.44)	1.71 (0.75)	115.8 (3.72)	12 (66.7)
	Week 8	81.6 (6.32)	97.8 (6.67)	28.6 (2.74)	108.7 (9.24)	30.6 (6.95)	4.83 (0.91)	1.20 (0.40)	1.78 (0.88)	128.9 (4.50)	15 (83.3)
BA (*n* = 18)	Week 0	76.5 (8.07)	93.9 (6.26)	26.6 (1.61)	106.7 (8.92)	28.9 (5.71)	4.72 (0.82)	1.27 (0.34)	1.66 (0.76)	105.8 (4.79)	8 (44.4)
	Week 4	73.1 (7.25)	91.6 (5.37)	25.9 (1.27)	103.8 (8.75)	27.2 (5.14)	4.25 (0.73)	1.31 (0.37)	1.45 (0.81)	122.8 (3.11)	13 (72.2)
	Week 8	71.1 (7.62)	90.8 (4.18)	25.6 (1.17)	102.5 (8.61)	26.9 (5.32)	4.06 (0.76)	1.33 (0.47)	1.26 (0.72)	145.8 (9.19)	16 (88.9)
LC (*n* = 18)	Week 0	81.5 (5.80)	98.2 (6.19)	28.6 (1.51)	108.4 (9.22)	30.5 (6.06)	4.80 (0.88)	1.22 (0.40)	1.81 (0.85)	107.0 (3.96)	8 (44.4)
	Week 4	75.2 (6.15)	92.4 (4.23)	26.6 (0.90)	104.3 (8.75)	26.2 (6.31)	3.78 (1.05)	1.30 (0.53)	1.26 (0.75)	122.1 (1.86)	14 (77.8)
	Week 8	72.2 (6.43)	91.0 (3.17)	24.2 (0.69)	102.1 (8.54)	24.2 (6.15)	3.32 (0.76)	1.34 (0.66)	1.07 (0.95)	140.3 (5.52)	17 (94.4)
Time effect	F	2.29	34.146	45.373	1.58	4.89	12.62	0.23	3.65	302.62	–
	*P*	0.116	<0.001	<0.001	0.216	0.011	<0.001	0.795	0.033	<0.001	
Group effect	F	10.85	6.71	19.30	2.35	3.38	17.47	0.13	2.66	171.22	–
	*P*	<0.001	0.002	<0.001	0.105	0.041	<0.001	0.878	0.080	<0.001	

BW, body weight; BMI, body mass index; WC, waist circumference; HC, hip circumference; Meth, methamphetamine; CON, general physical activity; BFP, body fat percentage; TC, total cholesterol; HDL-C, high-density lipoprotein–cholesterol; TG, triglyceride; BA, Baduanjin intervention; LC, Baduanjin with LC intervention. QOL–DA, Quality of life scale for drug addicts; VAS, Visual Analog Scale.

### Lipid metabolism parameters

**Figure 2:**
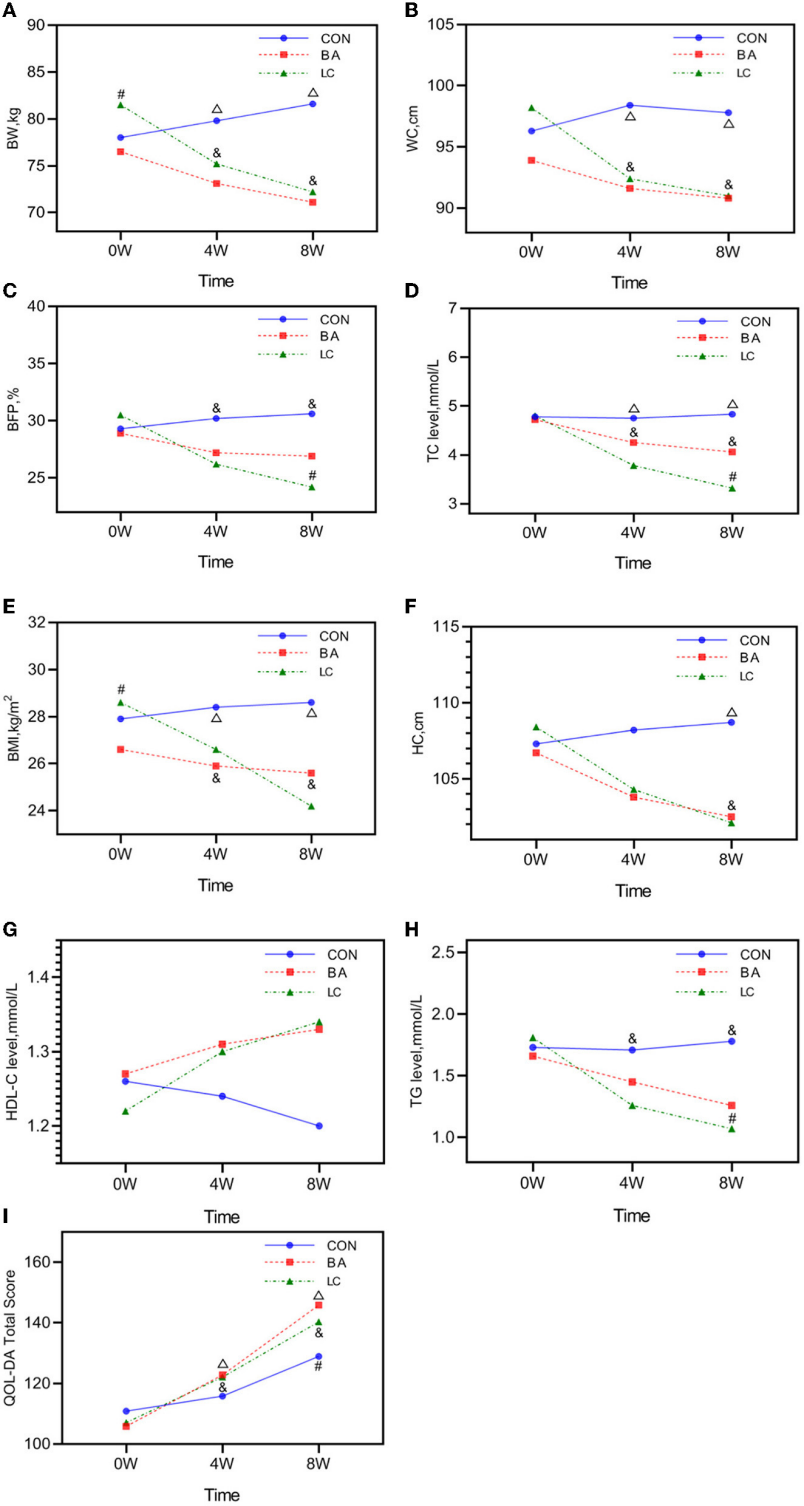
Analysis of the time–group interaction in the generalized estimating equation (GEE) models. **(A)** Time–group effect in the GEE model of BW. **(B)** Time–group effect in the GEE model of WC. **(C)** Time–group effect in the GEE model of BFP. **(D)** Time–group effect in the GEE model of TC level. **(E)** Time–group effect in the GEE model of BMI. **(F)** Time–group effect in the GEE model of HC. **(G)** Time–group effect in the GEE model of HDL-C level. **(H)** Time–group effect in the GEE model of TG level. **(I)** Time–group effect in the GEE model of QOL-DA. CON, routine rehabilitation exercise; BA, Baduanjin intervention; LC, Baduanjin combined with LC intervention; BW, body weight; BMI, body mass index; WC, waist circumference; HC, hip circumference; Meth, methamphetamine; CON, general physical activity; BFP, body fat percentage; TC, total cholesterol; HDL-C, high-density lipoprotein cholesterol; TG, triglyceride; BA, Baduanjin intervention; LC, Baduanjin with LC intervention; QOL–DA, Quality of life scale for drug addicts. Δ, difference between the BA and CON; #, difference between the BA and LC; &, difference between the LC and CON. *P* < 0.05.

The TC and TG levels significantly decreased in the BA and LC groups (*P* < 0.05), whereas no changes in the TC and TG levels occurred in the CON group (Table 2). However, the difference between the BA and LC groups was statistically significant at week 8 (*P* < 0.05; Figure 2). Although the HDL-C levels increased in the BA and LC groups, no significant difference in HDL-C was found between the three groups (*P* > 0.05; Figure 2).

### QOL-DA

A significant change in the group–time interaction was determined (*P* < 0.001), suggesting that the changing trend in QOL-DA scores varied depending on the intervention (Figure 2). Drug type exerted a significant effect on the change in scores (*F* = 5.924, *P* = 0.019). The QOL-DA scores of the LC group were slightly lower than those of the BA group at week 8 (*P* < 0.05; Table 2).

### VAS

With VAS scores distributed as the dependent variable (1 = <5 points, 0 = ≥5 points), if score <5 points, it was considered effective—that is, the craving was reduced (Table 2). The interaction term of intervention and time was considered the independent variable, and GEE analysis was conducted (Table 3). No significant differences in VAS score distribution (*P* > 0.05) or type of drugs (*P* > 0.05) existed between the three groups. The efficiency improved over time, and the craving decreased significantly at weeks 4 (*P* = 0.002) and 8 (*P* < 0.001). The predictive accuracy of the model was 72.84% (118/162).

**Table 3:** Analysis of VAS score distribution by generalized estimating equation.

**Parameter**	**B**	**OR (95%CI)**	***P-*value**
(Intercept)	2.275	9.72 (3.18–29.73)	<0.001
[Group = CON]	−0.309	0.73 (0.21–2.55)	0.626
[Group = BA]	−0.207	0.81 (0.25–2.62)	0.729
[Group = EX]	0		
[Type = Heroin]	−0.052	0.95 (0.32–2.86)	0.926
[Type = Meth]	0		.
[Time = Week 0]	−2.235	0.11 (0.05–0.25)	<0.001
[Time = Week 4]	−1.127	0.32 (0.16–0.66)	0.002
[Time = Week 8]	0		
(Scale)	1		

CON, general physical activity; BA, Baduanjin intervention; LC, Baduanjin with LC intervention; Meth, methamphetamine; VAS, Visual Analog Scale.

### Adverse events

No adverse events were reported in this trial.

## Discussion

Our study shows that for overweight patients, 4–8 weeks of Baduanjin intervention or Baduanjin intervention with LC can reduce body weight, BMI, and WC over time, indicating that both interventions can prevent the development of central obesity (WC ≥85 cm). These interventions can also improve the quality of life of patients in DRCs. We further found that compared with the Baduanjin intervention, the combined LC intervention provided additional benefits and thus more effectively reduced HC, BFP, TG, and TC levels. However, in DRCs, the Baduanjin intervention and general physical activity reduced drug craving, which was not relevant to the intervention measures. No adverse events were reported during the experiment. The data obtained show that Baduanjin, a mild physical and mental exercise, could be combined with LC diet therapy, effectively reducing BMI, controlling central obesity, reducing drug cravings, and improving the quality of life of patients. This study may contribute new ideas for our follow-up and other experiments related to exercise and diet control, healthy lifestyle promotion, and integrity maintenance among people who struggle with drug addiction (12).

The results of the present study show that after an 8-week intervention, the BW, BMI, WC, HC, and BFP of the BA and LC groups decreased. However, no change was observed in the CON group, suggesting that general physical activity was ineffective in weight loss. These findings are consistent with previous studies; the abdominal circumference of the Baduanjin group was also significantly improved after weeks 12 and 24 of the intervention (32). Study results on the beneficial effects of tai chi and TCE on reducing WC and body weight among obese older adults are also consistent (33, 34). A report contradicted the conclusion: although Baduanjin exercise reduces the accumulation of subcutaneous fat, it is not ideal for controlling abdominal obesity (35). The reason is that the BMI of the included participants was within the normal range, and the measurement results were affected by the time and period of intervention, the choice of exercise season, and whether the participants adopted a controlled lifestyle.

Given that reports have demonstrated and discussed the beneficial effects of Baduanjin on enhancing various health outcomes (36, 37), the following discussion focuses on the effects of (i) Baduanjin and (ii) combined Baduanjin and LC on observed variables (26, 27). Our findings showed that the combination of LC with Baduanjin effectively reduced BFP, TC, and TG. This effect was more evident in week 8, showing a continuous improvement in lipid metabolism. HDL-C in the LC group exhibited a gradual increase, but this change was not statistically significant.

A good fit and dietary habits are essential to overall health. Recent studies have shown that failure to maintain a healthy weight disrupts the metabolic–immune axis of the body, increasing the risk of deadly diseases such as COVID-19 and other infectious diseases (38). New evidence, mainly from Western countries, suggests that LC as a diet strategy can effectively manage several common and rare pathological conditions (13, 39–42). A meta-analysis indicated that adopting a low-carbohydrate diet is an effective and safe intervention for weight management in overweight and obese individuals (43). Consistent with our findings, all studies on low-carb diet intervention, except for those involving severely obese patients, have exhibited promising effects on weight loss and WC (average reduction: 1.5–14.3 kg and 2.2–9.3 cm, respectively) (44). Overweight and obese people showed an overall improvement in metabolic flexibility after weight loss. TC and TG levels decreased gradually with a reduction in BMI, whereas HDL-C levels steadily increased (45). TG and TC levels further decreased in the LC group, and TG and TC levels responded more favorably to the low-carb diet than to the conventional diet (46). This response could be related to the decrease in very-low-density lipoprotein triglycerides caused by reduced carbohydrate substrate transport in the liver and improved insulin sensitivity. However, caution is still necessary when recommending low-carb diets, as essential issues remain (47).

However, no significant changes in HDL-C were detected in our study, and the reasons for the differences have yet to be determined. A study in Korea revealed that HDL-C levels are affected by genetic factors and socioeconomic status (i.e., education, economic class, and occupation) (48). Emerging evidence suggests that HDL function is more important than quantity, and HDL-C may no longer be a reliable marker for cardiovascular diseases (49). Potential damage to vascular function arises from long-term drug abuse (50).

Exercise interventions are also becoming increasingly popular, owing to research findings that indicate effectiveness in treating substance abusers (51). Considering the physiological health benefits of LC combined with exercise, the subjective responses of patients to quality of life and cravings under LC conditions are necessary, given their importance for long-term adherence. To our knowledge, this is the first study to evaluate the effects of Baduanjin combined with LC intervention on the quality of life and drug craving response among overweight Chinese patients. Exercise is usually a social activity that promotes more social activities and contributes to overall mental health. Exercise can modulate inflammation, the synthesis and release of neurotrophins, and cerebral blood flow (52). Compared with teenagers, the long-term practice of Tai Chi can improve the memory performance of older women by remodeling the structure and function of the hippocampus (53). However, another study found that there are true interindividual differences in aerobic fitness and cognitive responses to aerobic exercise interventions in older adults (54). A meta-analysis of 19 studies showed that Baduanjin also contributes to quality of life, particularly in elderly individuals and patients with chronic diseases (55). A study on female meth addicts revealed that TCE participants have a lower recurrence rate than non-TCE participants (56). Baduanjin seems to exert a more lasting effect on reducing addiction. The reason is that mindfulness, typically consistent with deep rhythmic breathing, can help enhance discipline and activity endurance in an individual to achieve more extended periods of exercise (57). For the low-carb diet, the total carbohydrate intake was set to <130 g/day; to prevent ketosis, the lower carbohydrate intake limit was set to 70 g/day (58). With regard to adverse effects, a high-fat LC for 8 weeks may be detrimental to exercise performance, resulting in fatigue and a decline in quality of life (59). Obesity and illegal drugs have produced similar neurobiological adaptations (60–62). Food and drug intake activate dopamine neurons in the reward circuit; in addition, weight gain/obesity and chronic illegal drug use are associated with low expression of D_2_ dopamine receptors in the ventral striatum (63). Therefore, losing weight may help reduce drug craving. For this study, VAS reduction was mainly explained by the relatively closed environment and standardized treatment in DRCs.

Age may be one factor influencing the clinical outcomes of patients. The study did not analyse the difference in the effect of age; it is mainly due to the following reasons: the sample size, overweight participants, and the GEE model selected by statistical methods.

The strengths of this study include the well-controlled homogeneity of participants, the ability to measure before and after within the same time and place, and the monitoring of the Baduanjin intervention and dietary intake. The control group, representing general physical activity, was designed to ensure the effectiveness of different exercise intervention programs while excluding the effects of diet. However, several limitations exist. First, a large sample size should be used in a subsequent study, and further research is needed. Second, QOL-DA and VAS data on the participants were acquired *via* the self-report method in DRC, which may deviate from the actual social situation. Third, the findings are limited to overweight men, and whether they interact with age must be determined. Further research is needed on the mediating effects of drug type and years of drug use on weight loss. The intervention was limited to 8 weeks, which might be insufficient to demonstrate all of the protective effects. Future studies should include longer intervention durations and investigate willingness to continue exercise/dieting intervention based on the physical (weight loss and health improvements) and psychological (craving) returns from the intervention. The application of neuroimaging tools (e.g., fNIRS, functional MRI) or Electroencephalography can also be used to measure brain activity of drug addicts, and help deepen the understanding of the beneficial effects of the integrated program (64, 65).

Overall, the present study provides preliminary evidence of the potential effects of an 8-week Baduanjin intervention with LC on overweight people who struggle with drug addiction. Eight weeks of enhanced eating patterns (LC) and regular Baduanjin intervention may not only enhance anthropometric characteristics and body composition in overweight patients but may also positively affect other aspects, such as quality of life and mental health. Compared with Baduanjin exercise only, Baduanjin with LC intervention can more effectively reduce body fat and improve lipid metabolism and is equally safe. It may still be worth considering for patients undergoing LC interventions and continued rehabilitation after returning to society. In addition to practicing Baduanjin, patients should also learn to control their weight and maintain a healthy lifestyle. Moreover, the long-term safety and effectiveness of LC combined with regular exercise require further research.

## Data availability statement

The original contributions presented in the study are included in the article/supplementary material, further inquiries can be directed to the corresponding author/s.

## Ethics statement

The studies involving human participants were reviewed and approved by Ethics Committee of Zhejiang Police Vocational College. The patients/participants provided their written informed consent to participate in this study.

## Author contributions

DJ participated in the research design and coordination, conducted the experiments, and analyzed and interpreted the data. YX helped draft the manuscript. All authors have read and agreed to the published version of the manuscript.

## Funding

This work was supported by the 2022 Soft Science Research Program of Zhejiang Province (2022C35056).

## Conflict of interest

The authors declare that the research was conducted in the absence of any commercial or financial relationships that could be construed as a potential conflict of interest.

## Publisher's note

All claims expressed in this article are solely those of the authors and do not necessarily represent those of their affiliated organizations, or those of the publisher, the editors and the reviewers. Any product that may be evaluated in this article, or claim that may be made by its manufacturer, is not guaranteed or endorsed by the publisher.
